# SEFCA-Net: a semantic–edge–frequency cross-attention network for diabetic retinopathy lesion segmentation

**DOI:** 10.1117/1.JBO.31.10.106002

**Published:** 2026-10-08

**Authors:** Jin Xie, Jingtao Wang, Duo Xu, Yuzhong Peng, Kunlong Wu, Fangning Gu, Xue Zhang, Zhizhong Zhang, Xinjian Chen, Jianping Liao

**Affiliations:** a Nanning Normal University, Guangxi Key Lab of Human-Machine Interaction and Intelligent Decision, Nanning, China; b Nanjing University, School of Intelligence Science and Technology, Nanjing, China; c Soochow University, School of Electronics and Information Engineering, Suzhou, China; d Zhejiang Wanli University, College of Big Data and Software Engineering, Ningbo, China; e University of Electronic Science and Technology of China, School of Medicine, Chengdu, China; f Sichuan Provincial People’s Hospital, Eye Institute, Chengdu, China

**Keywords:** diabetic retinopathy, lesion segmentation, complementary feature fusion, semantic-guided window-based cross-attention

## Abstract

**Significance:**

Diabetic retinopathy (DR) lesion segmentation provides pixel-level lesion localization in fundus images. However, such lesions are often tiny, sparsely distributed, low-contrast, morphologically diverse, and visually similar to vessels or surrounding retinal structures, making accurate localization difficult. Many existing methods emphasize multi-scale spatial representation learning and handle boundary ambiguity and subtle texture differences mainly through encoder–decoder fusion. We therefore examine boundary and frequency representations as explicit, complementary inputs to semantic decoding.

**Aim:**

We aim to develop SEFCA-Net, a semantic–edge–frequency cross-attention network for diabetic retinopathy lesion segmentation. The main objective is to examine whether semantic, edge, and frequency-domain representations can be fused under semantic guidance to improve pixel-level lesion localization without compromising the semantic decoding stream. The evaluation focuses on four lesion categories, including hard exudates, hemorrhages, soft exudates, and microaneurysms, using public benchmarks and a DDR-to-IDRiD cross-dataset setting.

**Approach:**

SEFCA-Net uses HRNet-W48 as the semantic backbone to extract multi-scale semantic features and explicitly constructs edge and frequency-domain representations from input fundus images. The learnable multi-scale edge branch extracts boundary-sensitive features, while the all-channel multi-scale discrete cosine transform (DCT) frequency-domain branch generates texture-sensitive responses to low-contrast lesions. The semantic–edge–frequency cross-attention decoder uses the semantic representation as the primary stream and injects edge and frequency-domain features through semantic-guided window-based cross-attention. Performance is evaluated using the area under the precision–recall curve (AUPR), Dice, and Intersection-over-Union (IoU).

**Results:**

On DDR, SEFCA-Net achieves 47.85% mean AUPR (mAUPR), 46.76% mean Dice, and 31.22% mean IoU. On IDRiD, it reaches 69.74% mAUPR; when trained on DDR and tested on IDRiD, it obtains 59.83% mAUPR. Ablation studies show that either auxiliary branch improves the semantic-only baseline and that the complete configuration gives the highest mAUPR. Multi-scale Sobel modeling and DCT outperform the tested single-scale and wavelet alternatives, respectively.

**Conclusions:**

SEFCA-Net shows that semantic features can remain the main decoding stream while boundary-sensitive features and texture-sensitive responses are integrated in a controlled manner. Under the evaluated protocols, the model shows competitive in-domain performance and records the highest DDR-to-IDRiD mAUPR among the reproduced methods.

## 1 Introduction

Diabetic retinopathy (DR) remains one of the major causes of visual impairment worldwide.^1^^–^^3^ Color fundus photography is commonly used to assess retinal findings in DR, and deep learning systems have shown strong performance in image-level screening and referral decisions.^4^^–^^9^ DR lesion segmentation aims to localize lesions and quantify typical lesions, including hard exudates, hemorrhages, soft exudates, and microaneurysms. However, DR lesions are often tiny, sparsely distributed, low-contrast, and morphologically diverse, and some lesion regions are visually similar to vessels or surrounding retinal structures. Therefore, DR lesion segmentation remains a highly challenging task.

Current DR lesion segmentation methods mainly pursue stronger multi-scale spatial representation learning. L-Seg^10^ uses weighted multi-level feature fusion, whereas RTNet^11^ models intra-class lesion dependencies and inter-class lesion–vessel relationships. SAA,^12^ PMCNet,^13^ M2MRF,^14^ and HRDecoder^15^ further improve scale reweighting, cross-level consistency, feature reassembly, or high-resolution decoding. Although these methods are effective, and their central operations act on spatial feature maps; the separately parameterized edge and frequency branches examined here are not part of these designs.

High-resolution representations help preserve fine structures, while frequency-aware transforms can capture subtle texture changes and local details.^16^^,^^17^ However, separately derived boundary and frequency cues are not inherently reliable: gradients can respond to vessels, and frequency components may emphasize background variation. The resulting design question is how to integrate such features under semantic guidance.

This paper proposes SEFCA-Net, a semantic–edge–frequency cross-attention network designed for diabetic retinopathy lesion segmentation. The network uses HRNet as the backbone to generate multi-scale semantic features, integrates a multi-scale edge modeling branch to extract boundary-sensitive features, and introduces an all-channel multi-scale discrete cosine transform (DCT) frequency modeling branch to generate texture-sensitive responses. Semantic features are then used as the main decoding stream, while boundary-sensitive features and texture-sensitive responses are injected through semantic-guided window-based cross-attention. This asymmetric design aims to refine lesion localization while limiting interference from vessel edges and background frequency components.

The contributions of this work are mainly threefold. First, we formulate DR lesion segmentation as the fusion of semantic, edge, and frequency representations under semantic guidance and implement this framework in SEFCA-Net. Second, we design a learnable multi-scale edge branch, an all-channel multi-scale frequency branch, and a semantic-guided window-based cross-attention decoder to selectively integrate auxiliary information. Third, we evaluate the model through in-domain comparisons, a DDR-to-IDRiD transfer experiment, an efficiency analysis, and controlled ablations of the main design choices.

## 2 Related Work

### 2.1 Fundus Lesion Segmentation

Retinal lesion segmentation has evolved from methods targeting individual lesion types to networks that jointly predict several DR lesion categories. Early studies usually focused on a single lesion category or lesion group, such as red-lesion detection^18^ and exudate detection.^19^ A later dual-branch network with dual-sampling modulated Dice loss improved hard-exudate localization.^20^ Representative multi-lesion methods include L-Seg,^10^ which adopts weighted multi-level feature fusion; RTNet,^11^ which models intra-class lesion dependencies and inter-class lesion-vessel relationships; and SAA,^12^ which dynamically adjusts multi-scale feature weights through scale-aware attention. PMCNet,^13^ M2MRF,^14^ and HRDecoder^15^ further improve performance through progressive multi-scale consistency, many-to-many feature reassembly, and high-resolution decoding. MSAG combines multi-scale inputs with hierarchical spatial attention on DDR and IDRiD.^21^ Attention-DeepLab places convolutional block attention modules after the atrous spatial pyramid pooling module and in the low-level feature path of the DeepLabV3+ decoder to refine semantic responses and spatial details.^22^ SEFCA-Net instead explicitly constructs Sobel-derived edge and DCT-derived frequency representations and integrates them into an HRNet semantic stream through semantic-guided cross-attention. These studies demonstrate the value of improved spatial aggregation.

### 2.2 Multi-Scale and High-Resolution Representation Learning

Modern semantic segmentation performance is closely related to the quality of multi-scale and high-resolution representations, a trend also emphasized in broad medical image analysis surveys.^23^^,^^24^ FCN^25^ and U-Net^26^ establish the encoder–decoder paradigm for dense prediction. V-Net,^27^ U-Net++,^28^ and ResUNet-a^29^ further explore richer skip fusion and multi-scale refinement in medical or high-resolution settings. FPN^30^ and DeepLabV3+^31^ improve multi-scale context aggregation through pyramid fusion and atrous spatial pyramid pooling, respectively. HRNet^16^ maintains parallel streams at several resolutions and repeatedly exchanges information among them, preserving spatial detail while accumulating semantic context. Transformer-based models, starting from ViT^32^ and later developing into hierarchical variants such as Swin Transformer^33^ and SegFormer,^34^ continue this direction with stronger long-range context modeling. In fundus image analysis, M2MRF^14^ and HRDecoder^15^ confirm that refined multi-scale features and high-resolution decoding are important for tiny lesion segmentation. For SEFCA-Net, the remaining design question is how separately derived boundary and frequency features should update this high-resolution semantic stream.

### 2.3 Boundary and Frequency-Domain Auxiliary Modeling

Beyond semantic feature extraction, auxiliary structural and spectral information has also been explored in dense prediction. HED^35^ shows that explicit boundary-aware learning can improve pixel-level prediction, while CBAM^36^ demonstrates that lightweight spatial and channel attention can refine feature extraction by strengthening informative responses. In frequency-domain analysis, Wave-ViT,^17^ fast Fourier convolution,^37^ FsaNet,^38^ and LCSeg-Net^39^ show that spectral transforms or frequency-aware operators can preserve detailed features and improve segmentation, especially for low-contrast structures. These studies indicate that structural or spectral information can enhance the expressiveness of semantic features. In this paper, the phrase “complementary representations” refers specifically to the outputs of the HRNet semantic branch, the Sobel-derived boundary branch, and the DCT-derived frequency branch. This operational definition supports branch-level ablation and does not imply that other architectures cannot learn related cues implicitly.

## 3 Method

### 3.1 Overall Architecture

Figure 1 shows the overall architecture of SEFCA-Net. Given an input fundus image $I\in R^{3\times H\times W}$, the model first uses an HRNet-W48^16^ encoder to extract semantic features at four different resolutions, denoted as $F_{4}$, $F_{8}$, $F_{16}$, and $F_{32}$, with strides of 4, 8, 16, and 32, respectively. On the decoder side, these features are transformed by a 1×1 projection followed by spatial attention refinement into $C_{4}$, $C_{8}$, $C_{16}$, and $C_{32}$. Among them, $C_{4}$ preserves higher-resolution spatial details, while $C_{8}$, $C_{16}$, and $C_{32}$ are aligned to the same spatial resolution and fused to form the low-resolution semantic body.

**Fig. 1:**
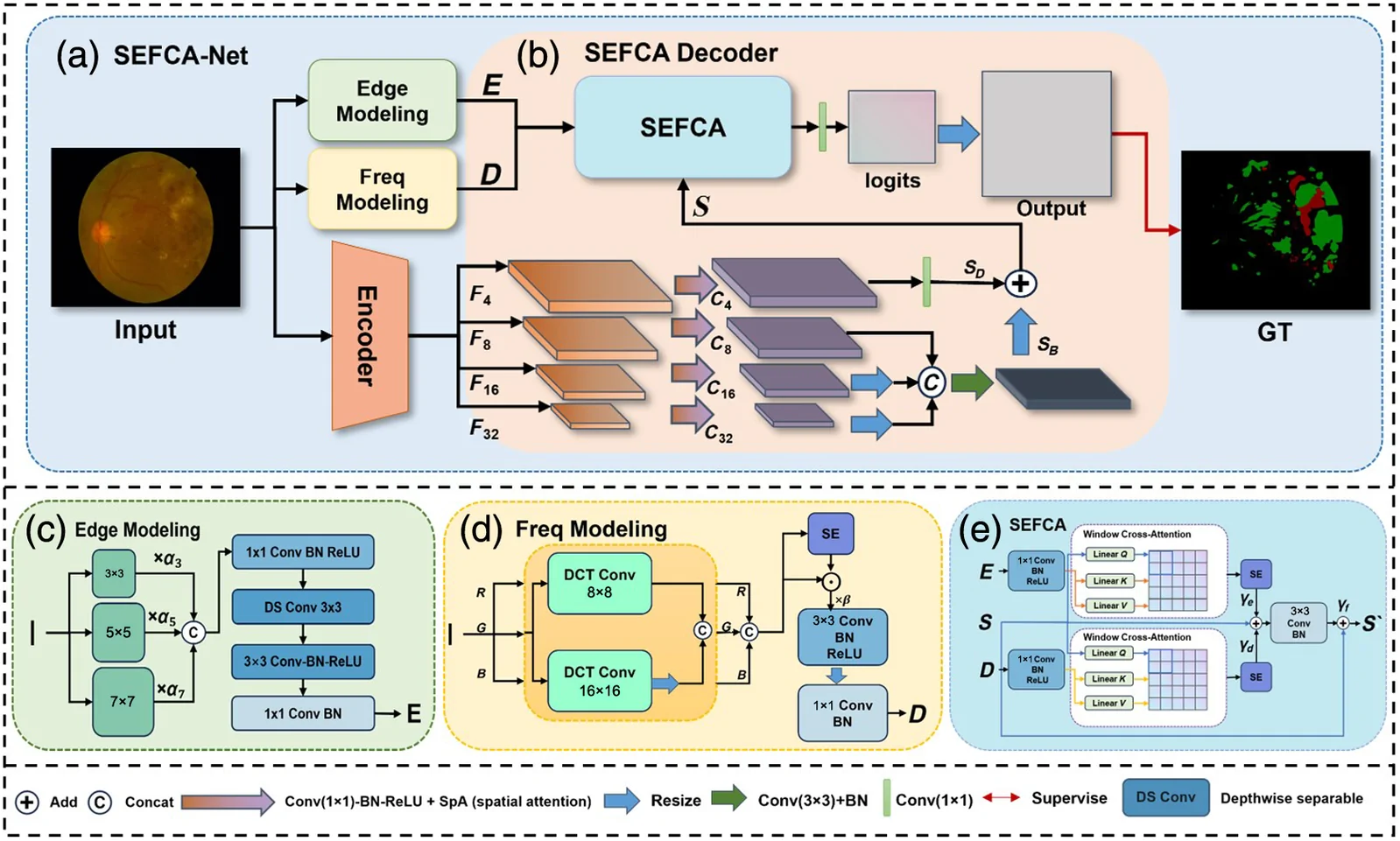
Architecture of SEFCA-Net. (a) Overall network. (b) Semantic decoder. (c) Learnable multi-scale edge branch. (d) All-channel multi-scale DCT branch. (e) Semantic-guided window-based cross-attention.

In addition to the semantic backbone, SEFCA-Net explicitly constructs two auxiliary representations, namely, edge and frequency-domain representations. The edge branch extracts multi-scale gradient features using 3×3, 5×5, and 7×7 Sobel kernels to supplement lesion boundary information. The frequency branch performs multi-scale DCT modeling on each color channel of the input image to obtain frequency information for low-contrast lesions. The semantic–edge–frequency cross-attention decoder then uses the semantic representation as the main stream, introduces edge and frequency features into the decoding process through semantic-guided window-based cross-attention, and outputs prediction logits for the four lesion classes. The implementation details of each module of SEFCA-Net are described in Secs. 3.2–3.5.

### 3.2 Learnable Multi-Scale Edge Modeling

A single fixed edge operator is difficult to adapt to the complex boundary patterns of fundus lesions, and the gradient scale suitable for describing these boundaries also varies. Therefore, we design a learnable multi-scale edge modeling branch, which introduces scale-adaptive weighting and lightweight convolutional enhancement on top of multi-scale edge information.

Specifically, as shown in Fig. 1(c), the branch applies Sobel kernels of sizes 3×3, 5×5, and 7×7 to obtain three groups of edge features, denoted as $M^{\left(3\right)}$, $M^{\left(5\right)}$, and $M^{\left(7\right)}$, respectively. Then, the softmax-normalized scale coefficients $\alpha_{3}$, $\alpha_{5}$, and $\alpha_{7}$ are used to adaptively weight the three scales, and the aggregated edge feature is fed into a lightweight fusion subnetwork. More specifically, a 1×1 convolution with batch normalization and ReLU adjusts the channel representation, a 3×3 depthwise separable convolution followed by a 3×3 convolution with batch normalization and ReLU enhances local boundary structures, and a final 1×1 convolution with batch normalization projects the feature to the decoder dimension. The final edge feature is written as

$$E=\mathrm{Align}({\mathrm{Proj}}_{e}\left({\mathrm{Enh}}_{e}\left(\mathrm{Cat}[\alpha_{3}M^{\left(3\right)},\alpha_{5}M^{\left(5\right)},\alpha_{7}M^{\left(7\right)}]\right)\right))\in R^{C_{e}\times H_{d}\times W_{d}},$$ (1)

where ${\mathrm{Enh}}_{e}(\cdot )$ denotes the edge fusion subnetwork in Fig. 1(c), which performs channel mixing and local structural enhancement, and ${\mathrm{Proj}}_{e}(\cdot )$ denotes the subsequent projection operation, which maps the fused feature to $C_{e}=32$ channels required by cross-attention. The resulting E preserves fine-grained edges, medium-scale contours, and coarse boundaries at the same time and can serve as an explicit boundary prior for the decoder.

### 3.3 All-Channel Multi-Scale Frequency-Domain Modeling

Frequency-domain modeling is used to supplement low-contrast lesion appearance differences that are difficult to distinguish from spatial features alone. Microaneurysms often appear as small local bright-dark changes, whereas exudates and hemorrhages are accompanied by broader intensity changes and texture differences. Different color channels also highlight different retinal structures. Based on this observation, we design an all-channel multi-scale frequency-domain modeling branch and use multi-scale DCT, SE channel recalibration, and frequency-band weighting to generate frequency-domain auxiliary features.

Specifically, as shown in Fig. 1(d), for each color channel c∈{R,G,B}, we use DCT convolution blocks with non-overlapping block sizes of 8 and 16 to encode two frequency scales. Each block produces four absolute coefficient maps. The convolution kernels are initialized with low- and mid-frequency DCT basis functions and are further optimized during training. The 16×16 responses are bilinearly upsampled to the 8×8 response resolution. After concatenating the frequency-domain features from all color channels at the two scales, we obtain the all-channel frequency-domain representation

$$D_{\mathrm{all}}=\mathrm{Cat}[{\left\{\Psi_{8}^{\left(c\right)}(I^{\left(c\right)}),\mathrm{Up}\left(\Psi_{16}^{\left(c\right)}(I^{\left(c\right)})\right)\right\}}_{c\in \{R,G,B\}}].$$ (2)

Finally, we first use a squeeze-and-excitation (SE) module to recalibrate the channels of $D_{\mathrm{all}}$ then apply the frequency band weights $\beta =\mathrm{softmax}(w_{\mathrm{freq}})$ to the 24 concatenated responses. A 3×3 convolution with batch normalization and ReLU fuses the weighted responses, and a 1×1 convolution with batch normalization produces the final feature

$$D=\mathrm{Align}({\mathrm{Proj}}_{d}\left({\mathrm{Fuse}}_{d}\left(\beta ⊙\mathrm{SE}\left(D_{\mathrm{all}}\right)\right)\right))\in R^{C_{d}\times H_{d}\times W_{d}},$$ (3)

where ${\mathrm{Fuse}}_{d}(\cdot )$ and ${\mathrm{Proj}}_{d}(\cdot )$ denote the 3×3 fusion block and 1×1 projection in Fig. 1(d), respectively. The projection sets the output channel dimension, while the decoder aligns the spatial resolution before cross-attention. The final D is a frequency-domain representation aligned with the decoder.

### 3.4 Semantic–Edge–Frequency Cross-Attention Decoder

The decoder head is responsible for introducing edge and frequency-domain information while preserving the semantic main stream. As shown in Figs. 1(b) and 1(e), the decoding process can be divided into two steps. First, a base semantic representation S is constructed from multi-scale semantic features. Then, S is used as the shared query feature to perform semantic-guided window-based cross-attention with the edge feature E and the frequency-domain feature D. This design keeps edge and frequency-domain information in a supplementary role and prevents them from replacing the dominant semantic branch.

Specifically, we first use the lightweight compression module ${\mathrm{Refine}}_{r}(\cdot )$, implemented as a 1×1 projection followed by spatial attention refinement, to process the semantic features at four scales and obtain $C_{4}$, $C_{8}$, $C_{16}$, and $C_{32}$. The low-resolution features are fused into the semantic body, while the highest-resolution feature is kept as detail guidance. This process is written as

$$C_{r}={\mathrm{Refine}}_{r}(F_{r}),\;r\in \{4,8,16,32\},\;S_{B}=\mathrm{Up}({\mathrm{Conv}}_{3\times 3}\left(\mathrm{Cat}[C_{8},\mathrm{Up}(C_{16}),\mathrm{Up}(C_{32})]\right)),\;S_{D}={\mathrm{Conv}}_{1\times 1}(C_{4}),\;S=S_{B}+S_{D}.$$ (4)

Here, $S_{B}$ and $S_{D}$ are the semantic body and detail guidance in Fig. 1(b), respectively, and S is the base semantic representation fed into the SEFCA module.

Then, we project the edge and frequency-domain features to the same dimension as S, partition them into non-overlapping local windows, and update S through two semantic-guided window-based cross-attention operations. With the default setting of w=16 and 4 attention heads, the process is written as

$$Y_{E}=\mathrm{WinMHA}(Q=S,K=V={\mathrm{Proj}}_{E}(E)),\;Y_{D}=\mathrm{WinMHA}(Q=S,K=V={\mathrm{Proj}}_{D}(D)),\;S_{\mathrm{aux}}=S+\gamma_{E}\mathrm{SE}(Y_{E})+\gamma_{D}\mathrm{SE}(Y_{D}),\;P_{\mathrm{logit}}=\mathrm{Head}(S+\gamma_{F}{\mathrm{ConvBN}}_{3\times 3}(S_{\mathrm{aux}})),$$ (5)

where WinMHA(·) denotes the local window multi-head cross-attention with relative position bias, ${\mathrm{Proj}}_{E}(\cdot )$ and ${\mathrm{Proj}}_{D}(\cdot )$ are the input projection layers that map the auxiliary features to the semantic-query channels, and Head(·) is the output head. $Y_{E}$ and $Y_{D}$ denote the edge-guided and frequency-guided semantic updates, respectively, and $\gamma_{E}$ and $\gamma_{D}$ are the LayerScale coefficients initialized to 0. After SE recalibration, the two updates are added to the base representation to form $S_{\mathrm{aux}}$. A 3×3 convolution with batch normalization transforms this feature, and a third zero-initialized LayerScale coefficient $\gamma_{F}$ controls its residual addition to the original semantic representation before Head(·). The window mechanism reduces global quadratic complexity to local window complexity and ensures that auxiliary information participates in updates only within semantically related local regions. During training and inference, the logits are finally resized to the input resolution and passed through a sigmoid function to output prediction probabilities.

### 3.5 Loss Function

The training objective uses a class-averaged binary Dice loss to address the severe class imbalance in fundus lesion segmentation. We apply sigmoid independently to each lesion channel and define the overall loss as

$$L=\frac{1}{K}\overset{K}{\underset{k=1}{\sum }}[1-\frac{2\underset{i}{\sum }p_{i}^{k}g_{i}^{k}+\varepsilon }{\underset{i}{\sum }{\left(p_{i}^{k}\right)}^{2}+\underset{i}{\sum }{\left(g_{i}^{k}\right)}^{2}+\varepsilon }],$$ (6)

where K=4 denotes the number of lesion categories; $p_{i}^{k}$ and $g_{i}^{k}$ denote the predicted probability and binary annotation of class k at pixel i, respectively; and $\epsilon =10^{-5}$ is the smoothing term. The background class is not included in the loss calculation.

## 4 Experiments

### 4.1 Experimental Setup

#### 4.1.1 Datasets

We evaluate SEFCA-Net on two public DR lesion segmentation benchmarks, DDR and IDRiD. IDRiD has been released both as a public dataset and as a lesion segmentation challenge benchmark,^40^^,^^41^ while DDR is widely used in several recent DR lesion segmentation studies.^14^^,^^15^^,^^42^ According to the data split used in the current experiments, DDR contains 383 training images, 148 validation images, and 225 testing images. IDRiD follows the official split and contains 54 training images and 27 testing images. Both datasets provide pixel-level annotations for four lesion categories: hard exudates (EX), hemorrhages (HE), soft exudates (SE), and microaneurysms (MA).

#### 4.1.2 Evaluation metrics

We use area under the precision–recall curve (AUPR), Dice, and Intersection-over-Union (IoU) as evaluation metrics and report the class-averaged results over the four lesion categories, namely, area under the precision–recall curve (mAUPR), mean Dice (mDice), and mean Intersection-over-Union (mIoU). Here, mDice is calculated from the evaluator’s mF1.^11^^,^^13^^,^^15^ Following the recommendation of M2MRF,^14^ we use AUPR as the primary metric because DR lesion segmentation has severe class imbalance and contains many tiny lesions. Dice and IoU are used as complementary measures of region overlap quality. Precision and sensitivity are summarized across thresholds by AUPR, while Dice corresponds to the binary F1 score at a fixed threshold. Specificity and area under the receiver operating characteristic curve (AUROC) are not additionally reported because they can be dominated by the large number of background pixels; lesion-level sensitivity and free-response receiver operating characteristic (FROC) require a separate connected component matching protocol beyond the pixel-level evaluation used here.

#### 4.1.3 Network settings

SEFCA-Net is implemented based on the MMSegmentation framework,^43^ and the semantic backbone is HRNet-W48.^16^ Unless otherwise specified, the edge branch outputs 32 channels, the frequency-domain branch outputs 8 channels, the decoder hidden dimension is 128, and the SEFCA module uses a default window size of 16 and 4 attention heads.

#### 4.1.4 Training details

All experiments follow a unified implementation and training protocol. DDR images are resized to 1024×1024 and trained for 40k iterations, while IDRiD images are resized to 1440×960 and trained for 20k iterations. The mini-batch size is 4 in all experiments. We use stochastic gradient descent (SGD) as the optimizer, with an initial learning rate of 0.01 and a weight decay of $5\times 10^{-4}$, together with warm-up and a poly learning rate decay schedule. During training, each image is randomly rescaled by a factor sampled from [0.5, 2.0], cropped to the target input size, horizontally flipped with a probability of 0.5, and randomly rotated within [−45 deg, 45 deg]. These geometric transformations form the base augmentation pipeline used in all main experiments. Validation and test images undergo deterministic resizing without random augmentation. Since IDRiD does not provide an official validation set, we do not perform additional hyperparameter tuning on its test set. Instead, we use the same training and tuning strategy as DDR and directly report test results under fixed settings. All locally reproduced comparison results and the ablations in Tables 5–10 are obtained from three independent runs, and the best result of each model or experimental setting is reported. Training experiments are mainly conducted on four RTX 4090D graphics processing units (GPUs). For models with higher memory consumption, training uses two A800 GPUs. Frames per second (FPS), parameter count, and giga floating-point operations (GFLOPs) in the efficiency evaluation are all measured on an RTX 5060 Ti. FPS is measured with a batch size of 1, 5 warm-up batches, and 200 timed batches per repeat, with 5 repeats.

### 4.2 Comparison with State-of-the-Art Methods

This section evaluates SEFCA-Net on DDR, IDRiD, and the cross-dataset setting from DDR to IDRiD. We reproduce DeepLabV3+,^31^ HRNetV2-W48,^16^ PSPNet,^44^ ConvNeXt-B,^45^ Swin-B,^33^ Twins-SVT-B,^46^ SegFormer-B5,^34^ and M2MRF-A^14^ under unified training settings and mark them with ‡. Published results for L-Seg,^10^ HED,^35^ DNL,^47^ SAA,^12^ PMCNet,^13^ RTNet,^11^ and MSAG^21^ are also included where available.

#### 4.2.1 Results on the DDR dataset

Table 1 presents the quantitative comparison on DDR. Among the locally reproduced baselines, SEFCA-Net achieves the highest 47.85% mAUPR, 46.76% mDice, and 31.22% mIoU. Compared with the second-best method, Twins-SVT-B,^46^ it improves the three mean metrics by 0.19, 0.35, and 0.38 percentage points. At the category level, SEFCA-Net achieves the best EX and SE AUPR and the second-best MA AUPR, Dice, and IoU among the reproduced methods.

**Table 1:** Quantitative comparison on the DDR dataset (%). ‡ denotes the methods evaluated under the unified settings; bold and italic data indicate the best and second-best results within this group, respectively.

Method	AUPR				mAUPR	Dice				mDice	IoU				mIoU
	EX	HE	SE	MA		EX	HE	SE	MA		EX	HE	SE	MA	
L-Seg	55.46	35.86	26.48	10.52	32.08	—	—	—	—	—	—	—	—	—	—
HED	61.40	43.19	46.68	20.61	42.97	56.63	42.61	45.50	22.43	41.79	39.50	27.09	29.46	12.63	27.17
DNL	56.05	47.81	42.01	14.71	40.14	53.36	42.71	40.40	15.60	38.02	36.39	27.15	25.33	8.46	24.33
SAA	62.69	44.56	37.47	19.33	41.02	—	—	—	—	—	—	—	—	—	—
PMCNet	54.30	39.87	31.64	19.94	36.44	—	—	—	—	39.31	—	—	—	—	32.29
MSAG	—	—	—	—	49.66	61.21	50.96	55.23	27.34	48.69	—	—	—	—	—
DeepLabV3+‡	63.26	45.58	*49.24*	15.76	43.46	58.10	44.73	49.31	23.32	43.86	40.95	28.80	32.72	13.20	28.92
HRNetV2-W48‡	63.82	51.68	46.97	18.76	45.31	57.57	49.39	47.20	25.69	44.96	40.42	32.79	30.89	14.74	29.71
PSPNet‡	56.84	53.11	44.28	9.16	40.85	51.90	49.30	44.62	15.22	40.26	35.05	32.71	28.72	8.24	26.18
ConvNeXt-B‡	56.62	47.90	49.15	8.93	40.65	49.85	30.52	*49.63*	14.27	36.07	33.20	18.01	*33.01*	7.68	22.98
Swin-B‡	60.20	50.92	44.17	14.85	42.54	53.27	43.12	43.77	22.10	40.56	36.30	27.49	28.02	12.42	26.06
Twins-SVT-B‡	*64.95*	**57.64**	47.08	**20.97**	*47.66*	57.09	**51.96**	48.37	**28.22**	*46.41*	39.94	**35.10**	31.90	**16.43**	*30.84*
SegFormer-B5‡	64.29	*57.14*	45.45	19.61	46.62	*60.05*	*50.06*	44.19	26.44	45.19	*42.91*	*33.39*	28.36	15.24	29.97
M2MRF-A‡	64.46	53.29	47.75	19.53	46.26	**60.07**	47.25	48.94	26.35	45.65	**42.93**	30.93	32.39	15.17	30.36
SEFCA-Net (ours)	**65.27**	53.66	**51.86**	*20.63*	**47.85**	58.53	49.89	**51.13**	*27.48*	**46.76**	41.38	33.24	**34.35**	*15.93*	**31.22**

#### 4.2.2 Results on the IDRiD dataset

Table 2 reports the results on IDRiD. SEFCA-Net obtains the highest mAUPR of 69.74% among the reproduced methods, slightly higher than SegFormer-B5^34^ at 69.73% and Swin-B^33^ at 69.49%. It also achieves the best EX Dice, EX IoU, and MA IoU among the reproduced methods.

**Table 2:** Quantitative comparison on the IDRiD dataset (%). ‡ denotes the methods evaluated under the unified settings; bold and italic data indicate the best and second-best results within this group, respectively.

Method	AUPR				mAUPR	Dice				mDice	IoU				mIoU
	EX	HE	SE	MA		EX	HE	SE	MA		EX	HE	SE	MA	
L-Seg	79.45	63.74	71.13	46.27	65.15	—	—	—	—	—	—	—	—	—	—
HED	80.81	66.41	68.09	40.45	63.94	78.60	64.33	67.00	38.81	62.18	64.74	47.43	50.38	24.07	46.66
DNL	75.12	64.04	64.73	32.48	59.09	73.15	61.87	63.96	32.78	57.94	57.67	44.80	47.03	19.61	42.28
SAA	88.12	67.04	72.81	41.52	67.38	—	—	—	—	—	—	—	—	—	—
PMCNet	87.24	67.05	71.11	46.94	68.08	—	—	—	—	56.02	—	—	—	—	43.12
MSAG	—	—	—	—	66.87	81.19	63.34	69.26	49.20	65.75	—	—	—	—	—
DeepLabV3+‡	86.14	70.75	72.48	40.07	67.36	79.64	64.71	69.00	38.67	63.00	66.16	47.83	52.67	23.97	47.66
HRNetV2-W48‡	85.65	67.69	**78.34**	40.89	68.14	78.72	62.81	**73.51**	43.85	64.72	64.90	45.79	**58.12**	28.09	49.22
PSPNet‡	77.47	69.98	74.39	33.31	63.79	71.63	65.54	70.70	36.23	61.03	55.80	48.75	54.68	22.13	45.34
ConvNeXt-B‡	85.73	66.02	71.89	**46.47**	67.53	78.83	60.45	67.95	45.99	63.31	65.06	43.32	51.46	29.86	47.43
Swin-B‡	*86.57*	**73.04**	75.32	43.05	69.49	78.88	**67.50**	70.81	45.25	**65.61**	65.12	**50.94**	54.81	29.24	**50.03**
Twins-SVT-B‡	82.51	64.27	*76.15*	42.46	66.35	75.87	61.33	71.29	44.80	63.32	61.12	44.23	55.39	28.87	47.40
SegFormer-B5‡	85.26	*71.98*	76.05	45.65	*69.73*	75.75	*67.18*	70.80	**47.54**	*65.32*	61.27	*50.73*	*56.01*	*30.19*	49.55
M2MRF-A‡	86.45	69.08	75.35	43.36	68.56	*79.69*	63.78	71.19	45.01	64.92	*66.23*	46.82	55.27	29.04	49.34
SEFCA-Net (ours)	**87.08**	69.83	75.99	*46.04*	**69.74**	**80.25**	62.13	*71.31*	*47.52*	65.30	**67.02**	45.06	55.41	**31.17**	*49.66*

#### 4.2.3 Generalization study

The cross-dataset experiment adopts the setting of training on DDR and testing on IDRiD, and the results are shown in Table 3. SEFCA-Net achieves the highest mAUPR of 59.83% among the reproduced methods and obtains the best AUPR of 79.44% for the EX category. Compared with the strongest reproduced baseline, Swin-B,^33^ mAUPR increases by 0.16 percentage points. Because DDR and IDRiD differ in lesion prevalence and image characteristics, the absolute mAUPR values in Tables 1–3 should not be compared across protocols.

**Table 3:** Cross-dataset generalization results from DDR to IDRiD (AUPR, %). ‡ denotes the methods evaluated under the unified transfer setting; bold and italic data indicate the best and second-best results within this group, respectively.

Method	AUPR				mAUPR
	EX	HE	SE	MA	
L-Seg	65.01	44.05	30.59	19.86	39.88
RTNet	67.99	45.04	34.01	21.14	42.05
DeepLabV3+‡	73.16	52.86	55.01	27.63	52.17
HRNetV2-W48‡	78.65	**64.36**	59.66	32.18	58.71
PSPNet‡	74.98	59.95	*66.73*	28.92	57.65
ConvNeXt-B‡	77.84	54.82	63.03	**35.12**	57.70
Swin-B‡	77.65	62.16	65.56	33.31	*59.67*
Twins-SVT-B‡	78.81	56.51	**66.99**	33.45	58.94
SegFormer-B5‡	*79.31*	62.72	61.32	33.23	59.14
M2MRF-A‡	78.23	61.30	62.74	33.52	58.95
SEFCA-Net (ours)	**79.44**	*62.76*	62.30	*34.81*	**59.83**

### 4.3 Efficiency Analysis

Table 4 compares the accuracy and computational cost. Except for HRDecoder, which uses 2048×2048 inputs, all other methods are evaluated at 1024×1024.^15^ SEFCA-Net achieves 47.85% mAUPR and 12.33 FPS. Compared with HRNetV2-W48, SEFCA-Net has a similar number of parameters and slightly lower GFLOPs while improving both mAUPR and FPS.

**Table 4:** Efficiency comparison. HRDecoder uses 2048×2048 inputs; all other methods use 1024×1024. Bold and italic data indicate the best and second-best results, respectively.

Model	Input size	mAUPR ↑	Params (M) ↓	GFLOPs (G) ↓	FPS ↑
HRNetV2-W48‡	1024×1024	45.31	65.85	375.00	*11.40*
DeepLabV3+‡	1024×1024	43.46	*60.21*	1017.00	6.30
PSPNet‡	1024×1024	40.85	65.60	1026.00	6.94
ConvNeXt-B‡	1024×1024	40.65	121.00	1166.00	6.19
Swin-B‡	1024×1024	42.54	120.00	1186.00	5.89
Twins-SVT-B‡	1024×1024	47.66	**43.16**	900.00	9.28
SegFormer-B5‡	1024×1024	46.62	81.97	420.00	6.69
M2MRF-A‡	1024×1024	46.26	70.36	**358.00**	11.13
SEFCA-Net (Ours)	1024×1024	*47.85*	65.86	*372.00*	**12.33**
HRDecoder‡	2048×2048	**48.67**	67.58	1955.00	6.39

### 4.4 Ablation Studies

The ablation experiments examine four aspects: the auxiliary branches and fusion strategy, the key settings of the edge and frequency-domain branches, the branch hyperparameters, and training time contrast augmentation. Since the task has clear class imbalance, the following analysis mainly focuses on mAUPR, with mDice and mIoU used as supporting metrics.

#### 4.4.1 Effects of auxiliary branches and fusion strategy

The ablation results of the auxiliary branches show that both edge and frequency-domain information can complement semantic decoding. When only the edge branch is added, mIoU and mDice are the best. When the full model introduces both the edge and frequency-domain branches, it achieves the highest mAUPR, indicating that the two auxiliary representations are complementary in terms of prediction reliability. The frequency-only setting also improves all three mean metrics over the semantic-only configuration (Table 5).

**Table 5:** Ablation results of auxiliary branches. The full model achieves the highest mAUPR, while the edge-only branch performs best on mIoU and mDice. Bold and italic data indicate the best and second-best results, respectively.

Semantic feature	Edge branch	Frequency branch	mIoU	mAUPR	mDice
✓			29.07	43.78	44.20
✓	✓		**31.43**	*46.84*	**46.91**
✓		✓	30.56	46.08	45.90
✓	✓	✓	*31.22*	**47.85**	*46.76*

The comparison of fusion strategies further shows that semantic-guided window-based cross-attention is more suitable for the current task than direct concatenation and co-attention. Its advantage lies in preserving the dominant role of the semantic branch while allowing edge and frequency-domain information to participate in updates in a controlled manner (Table 6).

**Table 6:** Ablation results of fusion strategies. Cross-attention achieves the best result on all three mean metrics. Bold and italic data indicate the best and second-best results, respectively.

Method	mIoU	mAUPR	mDice
Concat	30.06	*46.36*	45.37
Cross-attention	**31.22**	**47.85**	**46.76**
Co-attention	*30.36*	45.99	*45.70*

#### 4.4.2 Design analysis of edge and frequency-domain modeling

Edge scale and frequency-domain transform type are the two most direct module design choices. Table 7 shows that the multi-scale 3/5/7 setting outperforms all single-scale Sobel settings, suggesting that combining multiple gradient scales is beneficial for the boundary patterns represented in these experiments.

**Table 7:** Ablation results of Sobel scale settings in the edge branch. The multi-scale 3/5/7 setting is best on all three metrics. Bold and italic data indicate the best and second-best results, respectively.

Mode	Kernel (s)	mIoU	mAUPR	mDice
Single	3×3	31.04	46.73	*46.53*
Single	5×5	*31.07*	*47.08*	46.48
Single	7×7	30.05	45.51	45.30
Multi	3/5/7	**31.22**	**47.85**	**46.76**

Table 8 shows that DCT outperforms wavelet in the tested all-channel multi-scale frequency-domain branch. After replacing wavelet with DCT, mAUPR increases by 1.59 percentage points, and mIoU and mDice also improve by 0.30 and 0.52 percentage points, respectively.

**Table 8:** Ablation results of frequency-domain modeling methods. DCT outperforms wavelet transform on all three mean metrics. Bold data indicate the best result.

Frequency modeling	mIoU	mAUPR	mDice
Wavelet transform	30.92	46.26	46.24
DCT	**31.22**	**47.85**	**46.76**

#### 4.4.3 Analysis of branch hyperparameters

Tables 9 and 10 analyze the input scale and output capacity of the auxiliary branches, respectively. The 1024×1024 setting achieves the best mAUPR and is therefore selected as the default input scale. The 32/8 channel configuration is best on all 3 metrics; increasing the tested capacity to 64/16 does not provide a further gain.

**Table 9:** Ablation results of input resolution for the edge and frequency-domain branches. 1024×1024 achieves the highest mAUPR, while 256×256 is slightly higher on mIoU and mDice. Bold and italic data indicate the best and second-best results, respectively.

Input size	mIoU	mAUPR	mDice
256×256	**31.42**	*47.69*	**46.87**
512×512	30.94	46.62	46.25
1024×1024	*31.22*	**47.85**	*46.76*

**Table 10:** Ablation results of auxiliary branch output channels. The 32/8 configuration achieves the best results on all 3 mean metrics; both the smaller and larger tested configurations perform worse. Bold and italic data indicate the best and second-best results, respectively.

Channels	mIoU	mAUPR	mDice
16/4	28.16	44.80	42.93
32/8	**31.22**	**47.85**	**46.76**
64/16	*31.03*	*47.29*	*46.37*

#### 4.4.4 Effect of training-time contrast augmentation

All settings in Table 11 use the base geometric augmentation pipeline described in Sec. 4.1.4. The geometric-only setting adds no photometric transformation, whereas random gamma-L and random CLAHE-L are applied on top of this pipeline. Contrast-limited adaptive histogram equalization (CLAHE) denotes contrast-limited adaptive histogram equalization. Each added transformation is applied to the CIELAB lightness channel with a probability of 0.5; gamma is sampled from U(0.85,1.15), and the CLAHE clip limit from U(1.2,1.8). Validation and test images remain unchanged, and all other settings follow Sec. 4.1.4.

**Table 11:** Effect of training time contrast augmentation on DDR (%). All settings include the base geometric augmentation; “+” denotes an additional photometric transformation. Bold and italic data indicate the best and second-best results, respectively.

Training augmentation	mIoU	mAUPR	mDice
Geometric only	**31.22**	**47.85**	**46.76**
+ Random gamma-L	*30.89*	*46.34*	*46.29*
+ Random CLAHE-L	29.21	44.88	44.23

Neither added transformation improves the base geometric pipeline. Random gamma-L reduces mAUPR from 47.85% to 46.34%, and random CLAHE-L reduces it to 44.88%; mDice and mIoU follow the same ordering. MA AUPR also decreases from 20.63% to 18.16% and 16.65%, respectively. We therefore retain the base geometric augmentation pipeline for the main comparisons.

### 4.5 Qualitative Comparison

Figure 2 compares five regions of interest selected from DDR and IDRiD. The examples include small or low-contrast lesions, diffuse boundaries, vessel-adjacent structures, and variation in lesion scale and composition. Each row shows the full-image context, the cropped RGB region, ground truth, and predictions from HRNetV2-W48, M2MRF-A, SegFormer-B5, and SEFCA-Net. In several rows, SEFCA-Net preserves small or fragmented components that at least one baseline misses or merges; in Fig. 2(d), it avoids the isolated vessel region responses shown by the three baselines. These selected examples illustrate local behavior, while the aggregate comparison is reported in Tables 1 and 2.

**Fig. 2:**
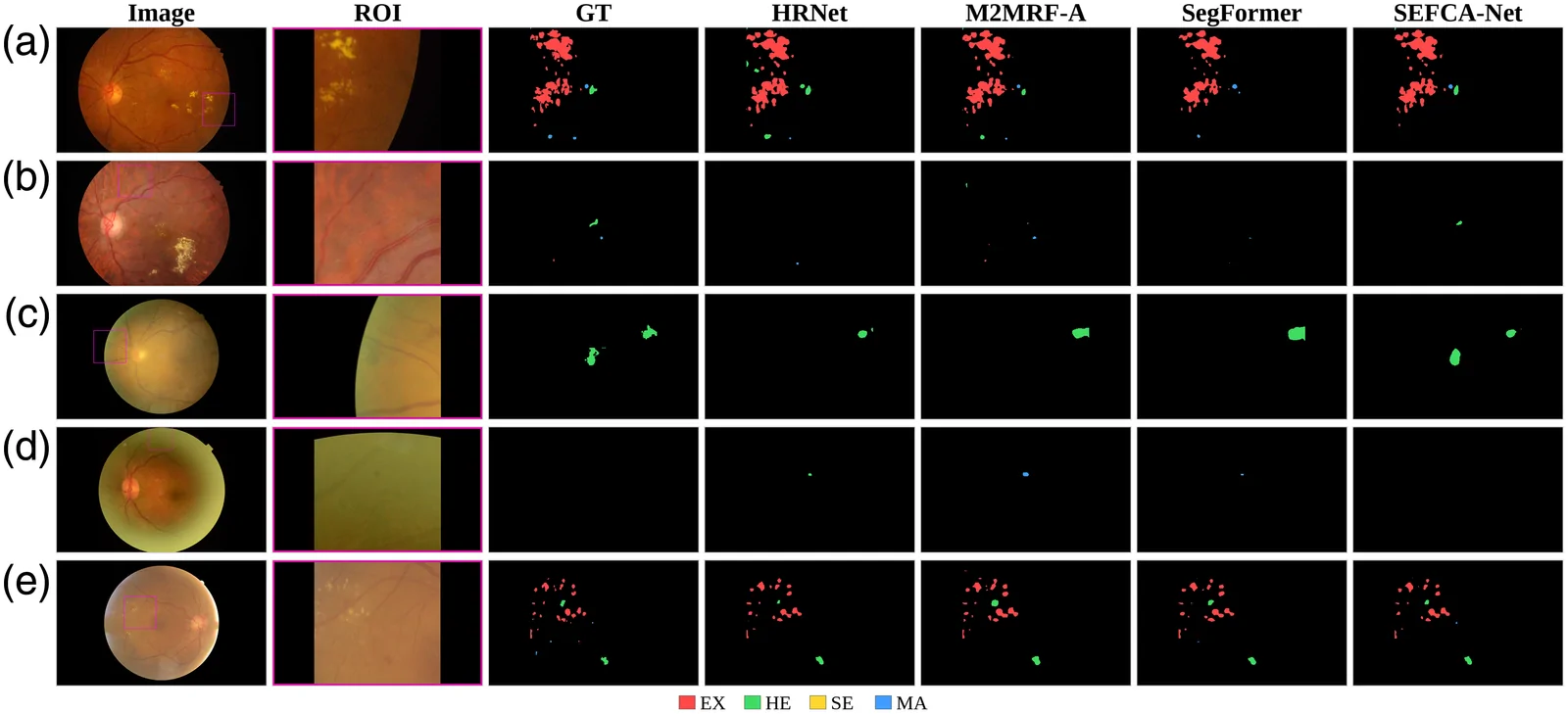
(a)–(e) Qualitative comparison on representative DDR and IDRiD cases.

## 5 Discussion and Conclusion

### 5.1 Discussion

The experiments indicate that SEFCA-Net improves the overall performance across lesion classes rather than dominating every metric. On the DDR dataset, the model achieves the best mAUPR, mDice, and mIoU under the unified reproduced setting. On the IDRiD dataset, its most evident advantage lies in mAUPR, while Dice and IoU remain close to those of strong baselines. This pattern supports competitive overall segmentation under class imbalance, but not uniform superiority across lesions or overlap measures.

This interpretation is supported by the ablation results. Within SEFCA-Net, a semantic-only branch must simultaneously encode contextual information, boundary features, and low-contrast texture features, which is particularly difficult for DR lesion segmentation. Adding the edge branch produces larger gains than adding the frequency branch alone on mAUPR, mDice, and mIoU. When the frequency branch is further combined with the edge branch, mAUPR increases from 46.84% to 47.85%, whereas mDice and mIoU remain slightly below the edge-only configuration. Thus, the ablation supports the contribution of both branches within the proposed network, without assigning a unique function to either branch.

The fusion study further shows that complementary information benefits from selective integration. Compared with direct concatenation and co-attention, semantic-guided window-based cross-attention achieves the best results on all three mean metrics. Within SEFCA-Net, this result suggests that edge and frequency features are more effective when they refine the semantic-dominant decoding stream than when they are concatenated or interact with it symmetrically.

SEFCA-Net also shows competitive transfer performance and efficiency under the evaluated settings. In the cross-dataset scenario where the model is trained on DDR and directly tested on IDRiD, SEFCA-Net achieves the best mAUPR among the reproduced methods. Although the improvement is limited, it suggests that the learned lesion representations are not completely restricted to a single data distribution. In addition, under the standard 1024×1024 resolution setting, SEFCA-Net achieves a higher mAUPR than HRNetV2-W48 and a faster inference speed while keeping the number of parameters almost unchanged. The efficiency result is specific to the hardware and timing settings described in Sec. 4.1.4.

At the same time, this study still has clear limitations. The evaluation is based on only two public DR lesion segmentation datasets and does not yet cover broader multi-center scenarios or pronounced device variability. Although SEFCA-Net achieves the best overall mAUPR among the reproduced methods on IDRiD, it is not consistently optimal on all metrics or lesion categories. Both edge and frequency information are derived from the same fundus image, so the method remains a single-source complementary representation framework rather than a solution designed for cross-device, multi-source, or temporal fundus analysis. Moreover, reporting the best of three runs does not quantify optimization variance, and the contrast study covers only two generic lightness transformations. Random gamma-L and random CLAHE-L do not improve SEFCA-Net under the tested ranges, but this result does not rule out photometric augmentation designed specifically for fundus images.

### 5.2 Conclusion

We propose SEFCA-Net for diabetic retinopathy lesion segmentation. The method combines HRNet-based semantic representation with learnable edge and frequency modeling and integrates them through semantic-guided window-based cross-attention while retaining the semantic representation as the main decoding stream. This design is intended to improve the representation of tiny lesions, blurred boundaries, and weak responses in low-contrast regions.

On the DDR and IDRiD datasets, SEFCA-Net shows competitive overall performance. On DDR, its mAUPR, mDice, and mIoU are all better than those of the reproduced baselines. On IDRiD, its mAUPR is also the best among the reproduced methods. In the DDR-to-IDRiD evaluation, the model again obtains the best mAUPR among the reproduced methods. Ablation results show that the auxiliary branches and the semantic-guided fusion strategy jointly improve model performance. These results support semantic–edge–frequency fusion as an effective design under the evaluated protocols. Future work will examine broader clinical validation, stronger cross-domain robustness, and lighter deployment-oriented variants.

## Data Availability

The source code, experiment configurations, model checkpoints corresponding to the reported point estimates, reference result files, and reproducibility instructions are publicly available at https://github.com/Judy-Jim/SEFCA-Net. The DDR and IDRiD datasets are not redistributed with the repository and should be obtained from their respective official sources.

## References

[1] Ting D. S. W. Cheung G. C. M. Wong T. Y., “Diabetic retinopathy: global prevalence, major risk factors, screening practices and public health challenges: a review,” Clin. Exp. Ophthalmol. 44(4), 260–277 (2016). 10.1111/ceo.12696 26716602

[r2] Thomas R. L. et al., “IDF Diabetes Atlas: a review of studies utilising retinal photography on the global prevalence of diabetes related retinopathy between 2015 and 2018,” Diabetes Res. Clin. Pract. 157, 107840 (2019). 10.1016/j.diabres.2019.107840 31733978

[3] Teo Z. L. et al., “Global prevalence of diabetic retinopathy and projection of burden through 2045,” Ophthalmology 128(11), 1580–1591 (2021). 10.1016/j.ophtha.2021.04.027 33940045

[4] Gulshan V. et al., “Development and validation of a deep learning algorithm for detection of diabetic retinopathy in retinal fundus photographs,” JAMA 316(22), 2402–2410 (2016). JAMAAP 1538-3598 10.1001/jama.2016.17216 27898976

[r5] Ting D. S. W. et al., “Development and validation of a deep learning system for diabetic retinopathy and related eye diseases using retinal images from multiethnic populations with diabetes,” JAMA 318(22), 2211–2223 (2017). JAMAAP 1538-3598 10.1001/jama.2017.18152 PMC5820739 29234807

[r6] Gargeya R. Leng T., “Automated identification of diabetic retinopathy using deep learning,” Ophthalmology 124(7), 962–969 (2017). 10.1016/j.ophtha.2017.02.008 28359545

[r7] Abramoff M. D. et al., “Improved automated detection of diabetic retinopathy on a publicly available dataset through integration of deep learning,” Invest. Ophthalmol. Vis. Sci. 57(13), 5200–5206 (2016). 10.1167/iovs.16-19964 27701631

[r8] Quellec G. et al., “Deep image mining for diabetic retinopathy screening,” Med. Image Anal. 39, 178–193 (2017). 10.1016/j.media.2017.04.012 28511066

[9] Rajalakshmi R. et al., “Automated diabetic retinopathy detection in smartphone-based fundus photography using artificial intelligence,” Eye 32, 1138–1144 (2018). 12ZYAS 0950-222X 10.1038/s41433-018-0064-9 PMC5997766 29520050

[10] Guo S. et al., “L-Seg: an end-to-end unified framework for multi-lesion segmentation of fundus images,” Neurocomputing 349, 52–63 (2019). 10.1016/j.neucom.2019.04.019

[11] Huang S. et al., “RTNet: relation transformer network for diabetic retinopathy multi-lesion segmentation,” IEEE Trans. Med. Imaging 41(6), 1596–1607 (2022). ITMID4 0278-0062 10.1109/TMI.2022.3143833 35041595

[12] Bo W. et al., “SAA: scale-aware attention block for multi-lesion segmentation of fundus images,” in Proc. IEEE Int. Symp. Biomed. Imaging (ISBI), pp. 1–5 (2022).

[13] He A. et al., “Progressive multiscale consistent network for multiclass fundus lesion segmentation,” IEEE Trans. Med. Imaging 41(11), 3146–3157 (2022). ITMID4 0278-0062 10.1109/TMI.2022.3177803 35613070

[14] Liu Q. et al., “Automated lesion segmentation in fundus images with many-to-many reassembly of features,” Pattern Recognit. 136, 109191 (2023). PTNRA8 0031-3203 10.1016/j.patcog.2022.109191

[15] Ding Z. et al., “HRDecoder: high-resolution decoder network for fundus image lesion segmentation,” Lect. Notes Comput. Sci. 15009, 328–338 (2024). LNCSD9 0302-9743 10.1007/978-3-031-72114-4

[16] Wang J. et al., “Deep high-resolution representation learning for visual recognition,” IEEE Trans. Pattern Anal. Mach. Intell. 43(10), 3349–3364 (2021). ITPIDJ 0162-8828 10.1109/TPAMI.2020.2983686 32248092

[17] Yao T. et al., “Wave-ViT: unifying wavelet and transformers for visual representation learning,” Lect. Notes Comput. Sci. 13685, 328–345 (2022). LNCSD9 0302-9743 10.1007/978-3-031-19806-9

[18] Orlando J. I. et al., “An ensemble deep learning based approach for red lesion detection in fundus images,” Comput. Methods Programs Biomed. 153, 115–127 (2018). CMPBEK 0169-2607 10.1016/j.cmpb.2017.10.017 29157445

[19] Prentasic P. Loncaric S., “Detection of exudates in fundus photographs using deep neural networks and anatomical landmark detection fusion,” Comput. Methods Programs Biomed. 137, 281–292 (2016). CMPBEK 0169-2607 10.1016/j.cmpb.2016.09.018 28110732

[20] Liu Q. et al., “Dual-branch network with dual-sampling modulated Dice loss for hard exudate segmentation in color fundus images,” IEEE J. Biomed. Health Inform. 26(3), 1091–1102 (2022). 10.1109/JBHI.2021.3108169 34460407

[21] Xu C. He S. Li H., “An attentional mechanism model for segmenting multiple lesion regions in the diabetic retina,” Sci. Rep. 14, 21354 (2024). SRCEC3 2045-2322 10.1038/s41598-024-72481-1 PMC11392929 39266650

[22] Jithesh A. et al., “Diabetic retinopathy lesion segmentation through attention mechanisms,” in Proc. IEEE Int. Conf. Mach. Learn. Appl. (ICMLA), pp. 808–813 (2025). 10.1109/ICMLA66185.2025.00118

[23] Litjens G. et al., “A survey on deep learning in medical image analysis,” Med. Image Anal. 42, 60–88 (2017). 10.1016/j.media.2017.07.005 28778026

[24] Shen D. Wu G. Suk H.-I., “Deep learning in medical image analysis,” Annu. Rev. Biomed. Eng. 19(1), 221–248 (2017). ARBEF7 1523-9829 10.1146/annurev-bioeng-071516-044442 PMC5479722 28301734

[25] Long J. Shelhamer E. Darrell T., “Fully convolutional networks for semantic segmentation,” in Proc. IEEE Conf. Comput. Vis. Pattern Recognit. (CVPR), pp. 3431–3440 (2015). 10.1109/TPAMI.2016.2572683 27244717

[26] Ronneberger O. Fischer P. Brox T., “U-Net: convolutional networks for biomedical image segmentation,” Lect. Notes Comput. Sci. 9351, 234–241 (2015). LNCSD9 0302-9743 10.1007/978-3-319-24574-4

[27] Milletari F. Navab N. Ahmadi S.-A., “V-Net: fully convolutional neural networks for volumetric medical image segmentation,” in Proc. Fourth Int. Conf. 3D Vision (3DV), pp. 565–571 (2016).

[28] Zhou Z. et al., “UNet++: redesigning skip connections to exploit multiscale features in image segmentation,” IEEE Trans. Med. Imaging 39(6), 1856–1867 (2020). ITMID4 0278-0062 10.1109/TMI.2019.2959609 PMC7357299 31841402

[29] Diakogiannis F. I. et al., “ResUNet-a: a deep learning framework for semantic segmentation of remotely sensed data,” ISPRS J. Photogramm. Remote Sens. 162, 94–114 (2020). 10.1016/j.isprsjprs.2020.01.013

[30] Lin T.-Y. et al., “Feature pyramid networks for object detection,” in Proc. IEEE Conf. Comput. Vis. Pattern Recognit. (CVPR), pp. 2117–2125 (2017).

[31] Chen L.-C. et al., “Encoder-decoder with atrous separable convolution for semantic image segmentation,” Lect. Notes Comput. Sci. 11211, 801–818 (2018). LNCSD9 0302-9743 10.1007/978-3-030-01234-2_49

[32] Dosovitskiy A. et al., “An image is worth 16x16 words: transformers for image recognition at scale,” in Int. Conf. Learn. Representations (ICLR) (2021).

[33] Liu Z. et al., “Swin Transformer: hierarchical vision transformer using shifted windows,” in Proc. IEEE/CVF Int. Conf. Comput. Vis. (ICCV), pp. 10012–10022 (2021).

[34] Xie E. et al., “SegFormer: simple and efficient design for semantic segmentation with transformers,” in Adv. Neural Inf. Process. Syst., Vol. 34, pp. 12077–12090 (2021).

[35] Xie S. Tu Z., “Holistically-nested edge detection,” in Proc. IEEE Int. Conf. Comput. Vis. (ICCV), pp. 1395–1403 (2015).

[36] Woo S. et al., “CBAM: convolutional block attention module,” Lect. Notes Comput. Sci. 11211, 3–19 (2018). LNCSD9 0302-9743 10.1007/978-3-030-01234-2

[37] Chi L. Jiang B. Mu Y., “Fast Fourier convolution,” in Adv. Neural Inf. Process. Syst., Vol. 33, pp. 4479–4488 (2020).

[38] Zhang F. Panahi A. Gao G., “FsaNet: frequency self-attention for semantic segmentation,” IEEE Trans. Image Process. 32, 4757–4772 (2023). IIPRE4 1057-7149 10.1109/TIP.2023.3305090 37594865

[39] Yuan H. Peng J., “LCSeg-Net: a low-contrast images semantic segmentation model with structural and frequency spectrum information,” Pattern Recognit. 151, 110428 (2024). PTNRA8 0031-3203 10.1016/j.patcog.2024.110428

[40] Porwal P. et al., “IDRiD: diabetic retinopathy-segmentation and grading challenge,” Med. Image Anal. 59, 101561 (2020). 10.1016/j.media.2019.101561 31671320

[41] Porwal P. et al., “Indian diabetic retinopathy image dataset (IDRiD): a database for diabetic retinopathy screening research,” Data 3(3), 25 (2018). 10.3390/data3030025

[42] Li T. et al., “Diagnostic assessment of deep learning algorithms for diabetic retinopathy screening,” Inf. Sci. 501, 511–522 (2019). 10.1016/j.ins.2019.06.011

[43] MMSegmentation Contributors, “MMSegmentation: OpenMMLab semantic segmentation toolbox and benchmark,” https://github.com/open-mmlab/mmsegmentation (2020).

[44] Zhao H. et al., “Pyramid scene parsing network,” in Proc. IEEE Conf. Comput. Vis. Pattern Recognit. (CVPR), pp. 2881–2890 (2017).

[45] Liu Z. et al., “A ConvNet for the 2020s,” in Proc. IEEE/CVF Conf. Comput. Vis. Pattern Recognit. (CVPR), pp. 11976–11986 (2022).

[46] Chu X. et al., “Twins: revisiting the design of spatial attention in Vision Transformers,” in Adv. Neural Inf. Process. Syst., Vol. 34, pp. 9355–9366 (2021).

[47] Yin M. et al., “Disentangled non-local neural networks,” Lect. Notes Comput. Sci. 12360, 191–207 (2020). LNCSD9 0302-9743 10.1007/978-3-030-58555-6

